# Ground beetles (Carabidae) in urban habitats of Kaluga City (Russia)

**DOI:** 10.3897/BDJ.10.e76100

**Published:** 2022-01-19

**Authors:** Victor Aleksanov, Sergey Alekseev, Maxim Shashkov

**Affiliations:** 1 State Budgetary Institution of Kaluga Region “Parks Directorate”, Kaluga, Russia State Budgetary Institution of Kaluga Region “Parks Directorate” Kaluga Russia; 2 Institute of Mathematical Problems of Biology RAS – the Branch of Keldysh Institute of Applied Mathematics of Russian Academy of Sciences, Pushchino, Russia Institute of Mathematical Problems of Biology RAS – the Branch of Keldysh Institute of Applied Mathematics of Russian Academy of Sciences Pushchino Russia

**Keywords:** broadleaved forests, gardens, grasslands, pitfall traps, Central Russia

## Abstract

**Background:**

Ground beetles (Carabidae, Coleoptera) are one of the most species-rich and well-studied insect families. However, the number of published datasets is disproportionately low against the biodiversity of this group. According to GBIF, only a fifth of the percentage of all published data covers ground beetles. This article describes a sampling-event dataset providing primary data on ground beetles collected in urban and suburban habitats in Kaluga, a typical central Russian city. We surveyed habitats of different land-use types and the extent and intensity of anthropogenic influence: yards, gardens, quarries, small urban woodlands, grasslands and riparian habitats. Carabids were collected by pitfall traps during most of the vegetative season (mostly from late April - early May to at least early October) for 13 seasons between 1994 and 2015. In total, the dataset contains 189 carabid species and 79,091 specimens. The dataset provides information about species composition and abundance, habitat distribution, seasonal and long-term dynamics of carabid beetles in environments of different degrees of urbanisation.

**New information:**

This dataset is the first sampling-event dataset about carabids in various urban habitats published through GBIF.

## Introduction

Ground beetles (Coleoptera, Carabidae) are a particularly popular model group of organisms for many kinds of ecological and environmental research, including studies of urbanisation (e.g. Klausnitzer and Richter 1983, Weller and Ganzhorn 2004, Magura et al. 2008, Niemelä and Kotze 2009, Schuett et al. 2018). The number of such papers has rapidly increased since the early 2000s (Magura and Lövei 2020). However, the results of these studies show inconsistent patterns of the effects of urbanisation on carabids. Generally, ground beetle assemblages in urban areas are species-poor, but sometimes researchers find high species richness and some rare species in urban and suburban habitats (Eversham et al. 1996, Zolotarev and Belskaya 2015, Belskaya et al. 2019). For Russian urban areas, there is quite a large array of carabid surveys (e.g. Dorofeev 1995, Sharova and Kiselev 1999, Eremejeva and Efimov 2006, Semenova 2008, Aleksanov et al. 2010, Zolotarev and Belskaya 2015, Aleksanov et al. 2019, Belskaya et al. 2019). However, the assessment of species diversity for different cities is rather complicated due to variation in sampling design in terms of sample plot sets and sampling methods. Typically ten traps per plot are often used to survey urban forests and parks (Niemelä et al. 2002, Semenova 2008, Niemelä and Kotze 2009), but sometimes eight (Weller and Ganzhorn 2004) or five (Deichsel 2006) traps were exposed. In urban grasslands, researchers have used six traps (Hartley et al. 2007) or have not mentioned trap numbers at all (Sharova and Kiselev 1999, Eremejeva and Efimov 2006). To understand patterns of formation of ground beetles assemblages in urban areas, we definitely need primary data. However, the above-cited Russian urban studies of carabids did not publish these. For Russia, a series of datasets on ground beetles from habitats of relatively low disturbance were published recently (Konakova and Kolesnikova 2018, Alekseev et al. 2021, Makarov and Sundukov 2021, Sundukov and Makarov 2021, Zinovyev et al. 2021). Most of them are located in Nature Reserves and National Parks. A considerable dataset was published for broadleaved forests of Kaluga Oblast, including Kaluga Urban Okrug (Shashkov et al. 2020a, Shashkov et al. 2020b). The dataset presented here complements the above-cited datasets with information from highly transformed habitats of this region.

Kaluga is a typical Central Russian town, amongst which there are both provincial centres such as Tver, Vladimir or Tula and municipalities - Serpukhov, Kolomna or others. Such a city usually occupies areas ranging from dozens to just over 150 km^2^ and hosts populations of 100 to 400 thousand people. The centuries-old history and location, usually on a large river (Oka or Volga, for example) , result in an irregular planning and complex development history of such cities. The city centres usually formed between more than three and two hundred years ago, often spontaneously. Multi-storey housing can be surrounded by quarters of private householding with gardens and orchards. Wastelands with dense wild grass are usual, on the periphery of industrial zones, adjacent to railroads or even amongst high buildings, as a consequence of abandoned former Soviet projects. A large watercourse with its tributaries forms a complex mosaic of near-water habitats.

## Sampling methods

### Study extent

Kaluga City is situated in the west of European Russia, in its middle (non-Chernozem) zone on the Oka River 150 kilometres (93 mi) southwest of Moscow. The climate is moderately continental with distinct seasons: warm and humid summers and cold winters with stable snow cover. According to nearest (~ 70 km SW) weather station, for which open data are available - Suhinichi (RSM00027707), the average annual air temperature during years of investigation (1994-2015) was 5.8°C. The average temperature in July was +19.1°C and in January, −6.9°C. Annual precipitation was about 633 millimetres (Bulygina et al. 2014). The city is situated on the southern edge of a mixed broadleaved-coniferous forests subzone or continental biogeographical region (Anonymous 2016), on the north margin of the Central Russian Upland. The area of the City is 168.8 km^2^ and the population is about 330 thousand people.

Prevailing landscapes of Kaluga City are flat, with undulating moraine plains shaped by the Moscow stage of the Dnieper glaciation. The main type of sediments is postglacial mantle-loams. Watersheds are flat and poorly drained. The minimal height above sea level is 116-120 m and the highest point reaches 235 m a.s.l. Along the Oka River, there is a highly-dissected erosional plain.

**Figure 1.**
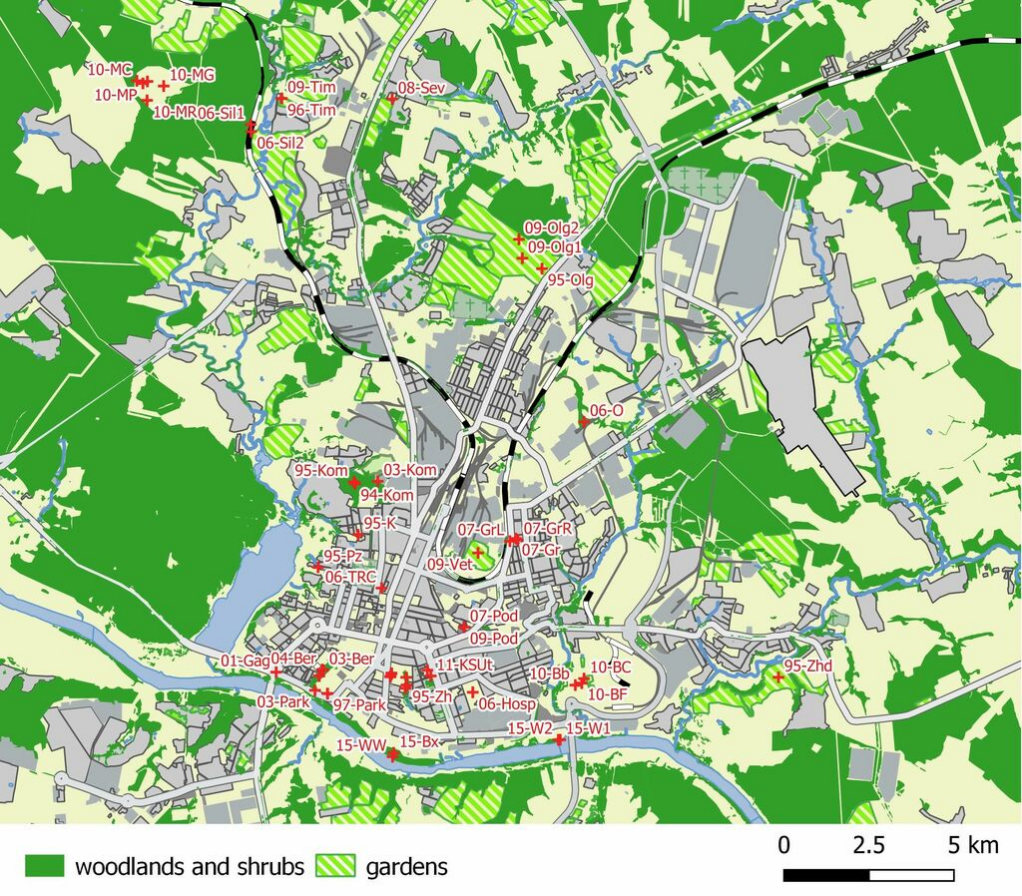
Location of surveyed habitats in Kaluga City and vicinities. Plot codes are the same as in Table 1 and correspond to parentEventID in the dataset.

Regarding vegetation zonation, the area belongs to the subzone of spruce-broadleaved forests, a spruce-oak vegetation district (Pashkang 1992). As for the typical central Russian provincial centre, the urban landscapes of Kaluga City have developed more or less smoothly since the 16^th^ century. The planning structures of Kaluga City were generally established during the last half of 18^th^ and the first half of the 19^th^ century. The modern city area has a striped pattern of residential and industrial buildings and agricultural lands because historically residential areas were planned near factories and other industrial objects. We distinguished three positions in the urban landscapes: city centre, city periphery and suburban zone. Locations of sample plots are mapped in Fig. 1. A brief characteristic of the sites is given in Table 1, including information on how the sample plots relate to city positions.

**Figure 2.**
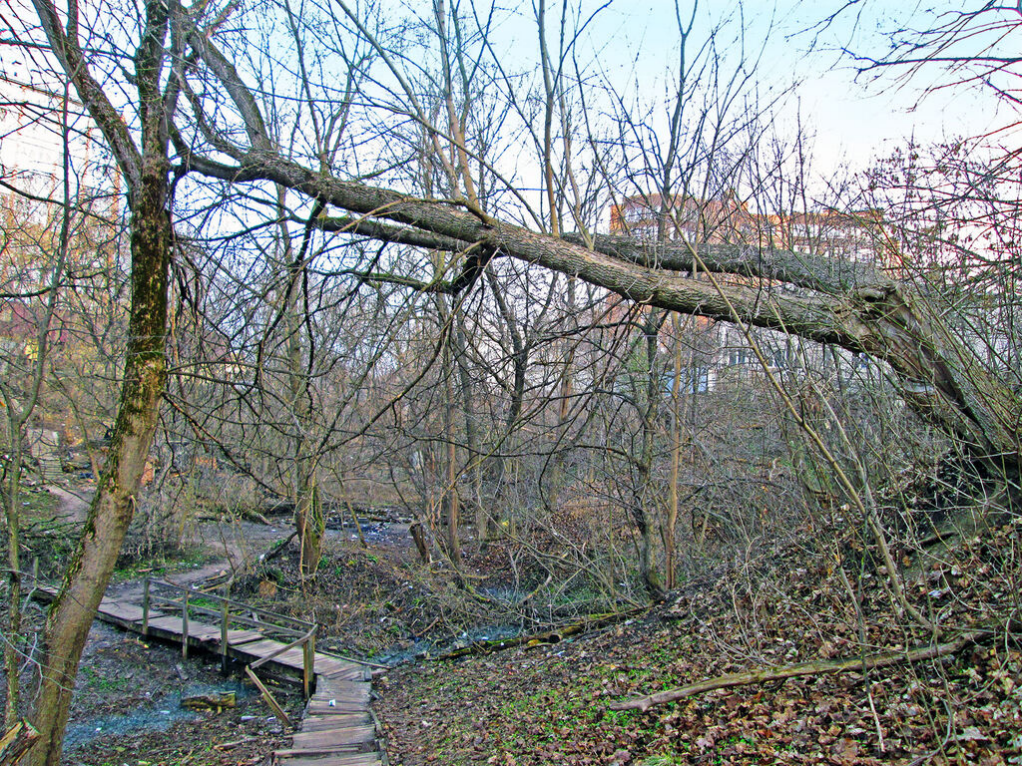
Small deciduous wood in a gulley (Zh).

**Figure 3.**
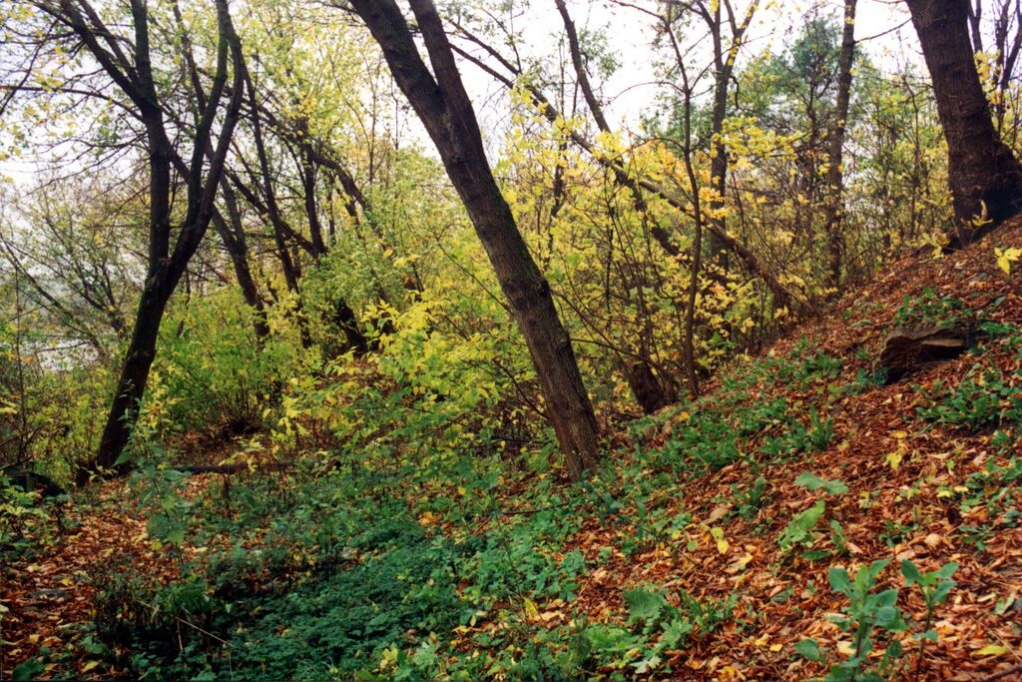
Small deciduous wood (03-Park)

**Figure 4.**
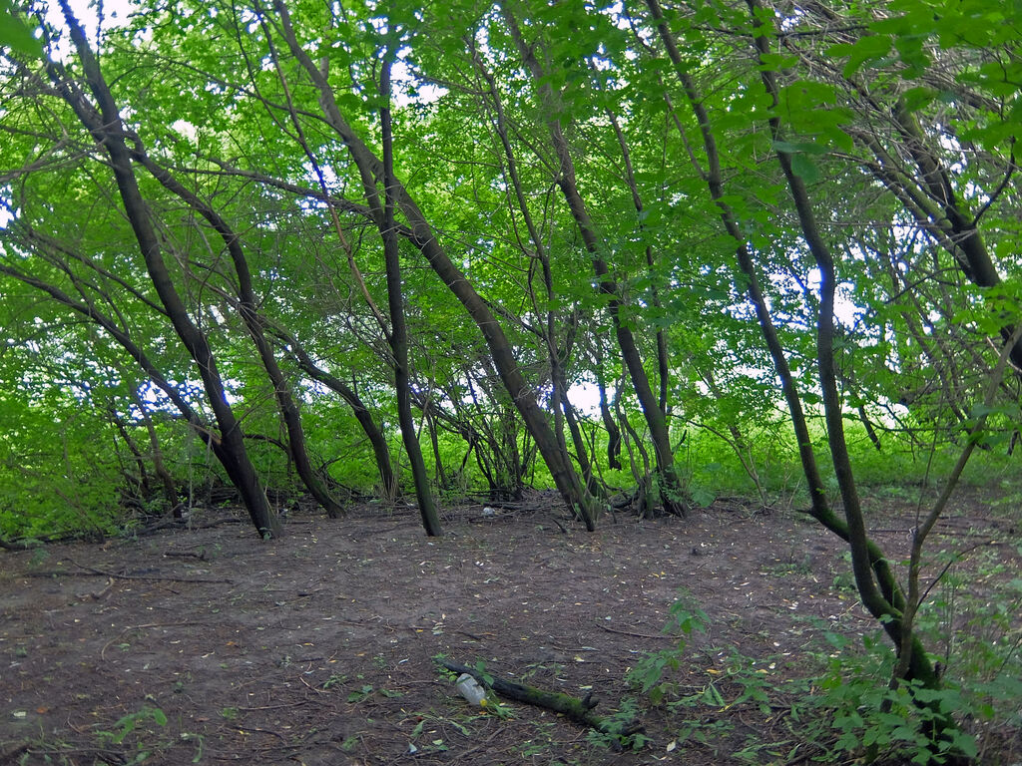
Box-elder (*Acernegundo*) spinney on the bank of Oka River (Bx).

**Figure 5.**
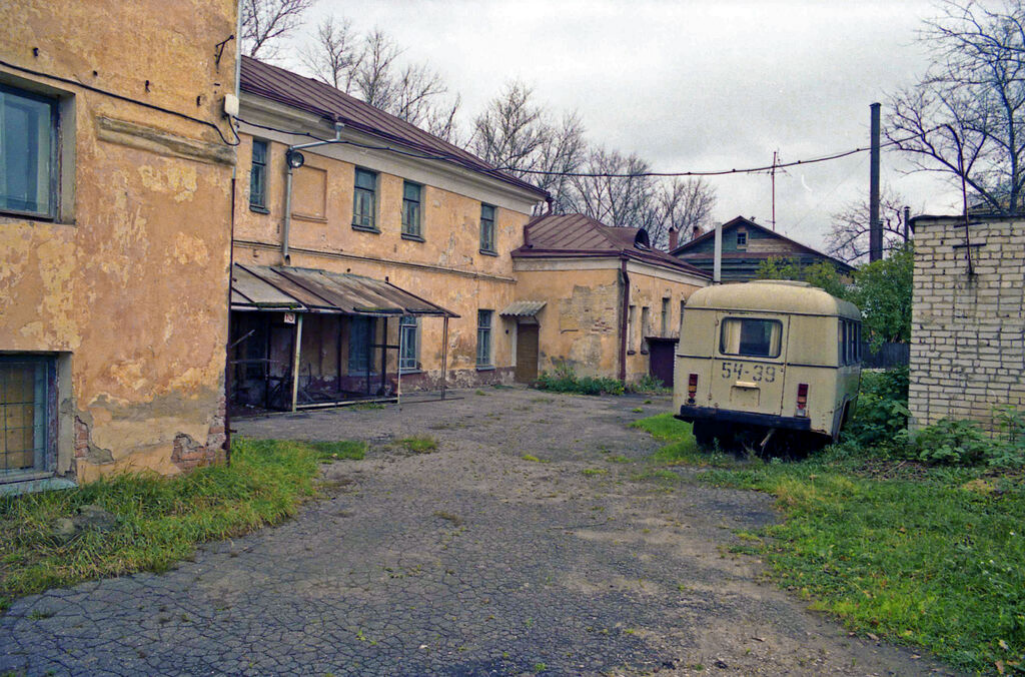
Yard with grass patches (EBCp).

**Figure 6.**
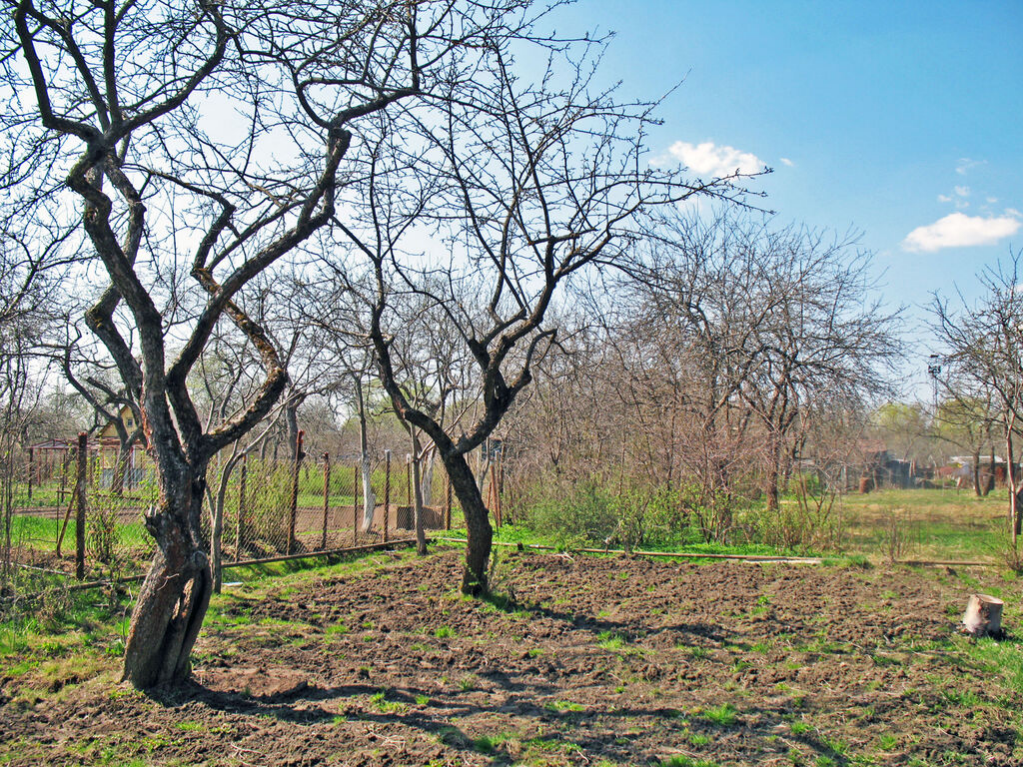
Garden (09-Vet).

**Figure 7.**
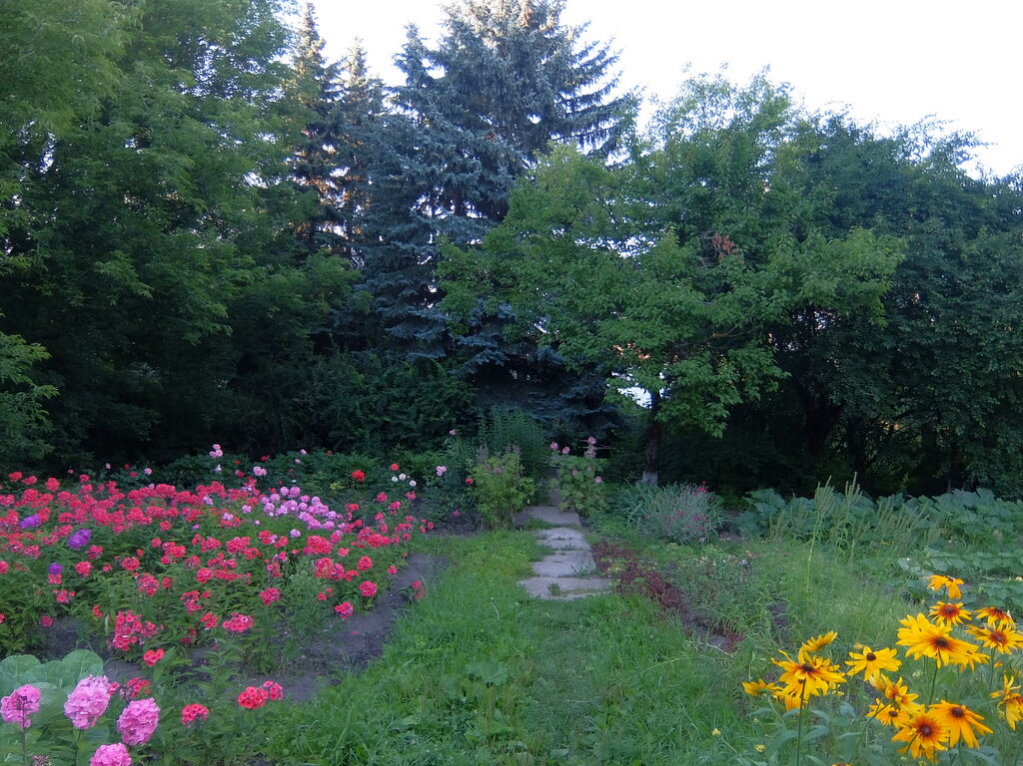
Garden (EBCg)

**Figure 8.**
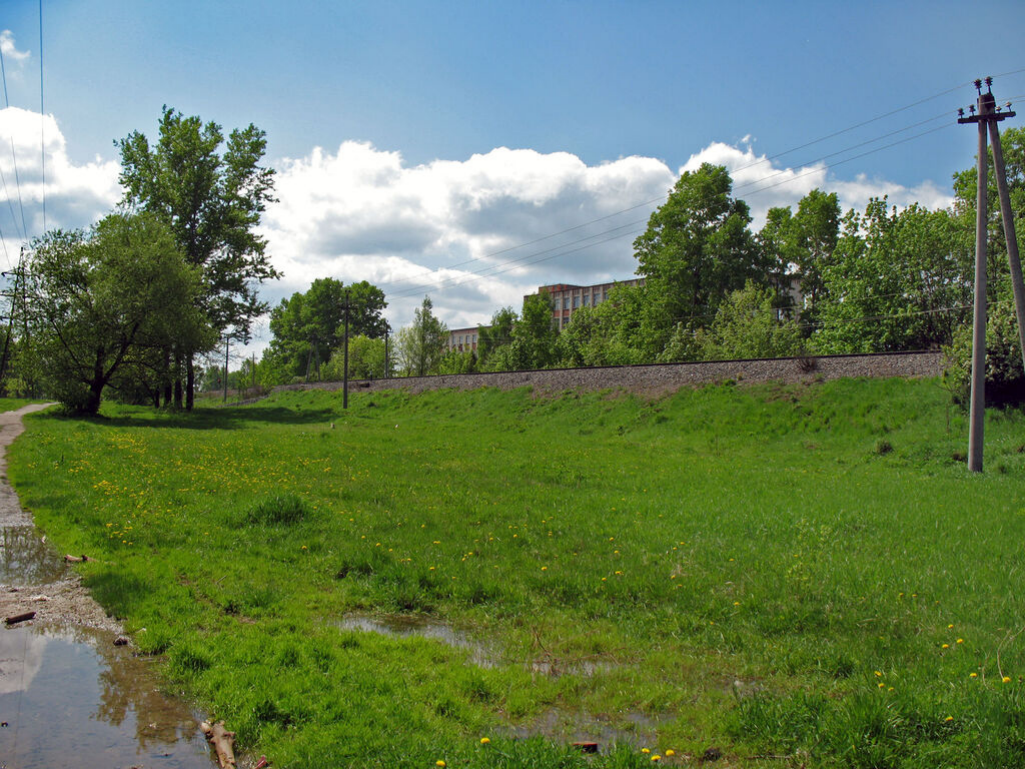
Grassland between a railway and a road (Gr).

**Figure 9.**
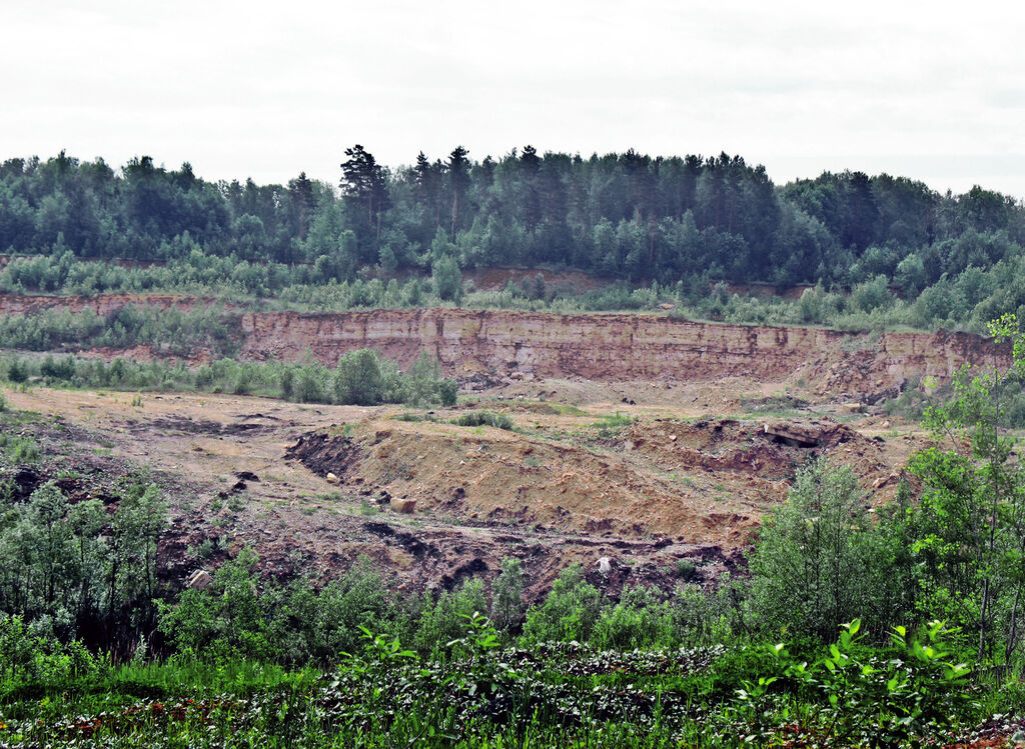
Limestone quarry.

Investigated sites can be grouped into six types of habitats which are characteristic of the urban area:

1. ***Forests*** (Fig. 2, Fig. 3) – habitats with area >0.5 ha where the dominant vegetation is trees with a canopy cover of at least 10%. In Kaluga, such habitats are located mainly in gullies and ravines. These sites are slightly managed and anthropogenic impact manifests mainly as littering. These forests are deciduous with *Acerplatanoides*, *Tiliacordata*, *Quercusrobur*, *Acernegundo*, *Ulmus* spp., *Populus* sp. and *Salix* spp. The herb layer is mainly shaped by nitrophilous weeds or nemoral herbs, which are stress-tolerators or ruderals. In one forest, the dominant tree was *Pinussylvestris* and, in another, it was silver birch (*Betulapendula*).

2. ***Riparian wooded habitats*** (Fig. 4) – sites along river, shaped by *Salixtriandra* and other small *Salix* spp. or box-elder (*Acernegundo*). The herb layer is mainly shaped by ruderal weeds and locally, there are deadcover patches. Formally, they can be considered as a forest, but they have some distinguishing habitat features: they are very narrow (about 20 m) and strongly impacted by the river. Riparian species have a large proportion in the ground beetle assemblages. Therefore, we distinguished this habitat as a distinct type.

3. ***Yards*** (Fig. 5) – building areas with lines of trees, ornamental gardens and small parks beside houses or in city squares. These habitats consist of small groups of trees, grassy patches, flowerbeds surrounded by buildings and pavement with artificial surfaces.

4. ***Gardens*** – habitats with a mosaic of cultivated trees and shrubs (mainly fruit) and herbs (vegetable or ornamental) without large buildings, roads and pavements (Fig. 6,Fig. 7). They include kitchen and allotment gardens. Soils are regularly tilled and irrigated. In Kaluga, gardens are aggregated to more or less large arrays. Some plots were fallow and were overgrown by ruderal herbs in the year of sampling.

5. ***Grasslands*** (Fig. 8) – in Kaluga City, grasslands are located mainly in wastelands between industrial buildings and protected belts along roads and railways. Sometimes, there are poor sites with *Calamagrostisepigeios* and, sometimes, there are plots dominated by mesophile grasses (*Festucapratense*, *Dactylus glomerata, Phleumpratense*); sometimes, there are poorly-drained sites with hygrophilic grasses and sedges.

6. ***Former quarry*** – the set of the biotopes on the slopes and bed of limestone the quarry, finally abandoned at least 30 years ago. The surveyed quarry is located in the northwest suburb and surrounded by spruce and pine forests. There is a village about one kilometre towards the north (Fig. 9). This type of habitat provides the possibility to investigate primary succession in vegetation and soil fauna population. That is why we consider this place as a distinct type of area.

### Sampling description

The beetles were collected with soil pitfall traps (0.5 l transparent plastic cups with a mouth of 85 mm in diameter filled to about a third (150 ml) with 4% formalin solution, with covers made of transparent polyethylene film). For the broadleaved forest of the Kaluga Region, we suggested that it needs 30 traps to reveal the species composition of carabids (Alexeev and Aleksanov 2017). Urban habitats are small and frequently disturbed, so we usually expose 15 or 10 traps for small plots. Some neighbouring plots were divided into two habitats after collecting the samples, so trap numbers for each habitat were fewer. Sometimes traps were destroyed by people. Therefore, the number of traps was fewer than 15 or 10 in such cases. For relatively large forests, 30 traps were exposed in some years. Pitfall traps were exposed continuously from April or May to October or November. In some cases, traps were operated for a shorter time, about two or three months (4 plots) or even about one and half months (1 plot, 01-Gag). For most samples, the traps were emptied within an interval of one to three weeks in most cases. Sometimes, the interval was longer, usually at the late season when the activity of carabids was low. Amongst 47 sampling plots, most were sampled once, i.e. during one season, six during two or three seasons and one during six. There were two consecutive seasons (no more) in five cases.

It is worth noting that plots with the same alphabetic acronym in code could be a different biotope (94-Zh, 95-Zh and 03-Zh) or similar biotopes in different, but places situated nearby (97-Ber and 03-Ber). Although such biotopes represented one continuous vegetation area within the same mesorelief form (afforested gullies, for instance), these may be different parts of it.

Thereafter, a series of continuous sampling events within one sampling plot during one season we called a "survey". We investigated 47 habitats (sample plots). Some of them were sampled during two, three or even six seasons. So, a total of 60 surveys were done. Unique values of DwC term parentEventID correspond to a distinct survey.

On each plot within a survey, 4 to 30 traps were established at the beginning of the season, but more often, 15 or 10 (less often). Usually, we chose sites for sampling within private (with the consent of owners), restricted (office territory) or low-attandance areas to ensure non-disturbance of the traps and the continuity of the investigations. Nevertheless, there were some cases of vandalism or unintentional destruction during lawn mowing, building repairing or accidental trampling when someone walked through the site. Trap flooding in the riparian sites has occurred as well. The event table in the DwC archive contains the actual traps number (intact ones) for each sampling event (dwc: samplingEffort). We tended to set the number of traps in multipliers of 5 or 10, but in some cases, the installation of new traps to replace the damaged ones was not possible, because of which the line of traps in a particular plot was shortened. In some sample plots (07-EBCg, 07-GrR, 09-Vet), traps were added after the first sampling when vegetation development has shown that installed traps did not cover the full diversity of the site.

So, in some cases, consecutive sampling events within one survey were based on different amounts of the traps. Dealing with the relative abundance (activity-densities) of carabids, we have consdered our data consistent and comparable with others datasets. When traps were disturbed, the seasonal sum of sampling efforts does not relate to the sampling duration as an integer value (Table 3).

Samples were sorted for carabids in the laboratory. Numerous and easily-recognisable species collected in 2003-2015 were identified by Victor Aleksanov. Specimens of those species, which were difficult to determine and all specimens collected before 2003, were identified by Sergey Alexeev. For identification, we used the following keys: Gureva and Kryzhanovskii (1965), Mandl (1983), Angus et al. (2001), Isaev (2002), Freude et al. (2004), Arndt et al. (2011). Identification of some doubtful specimens was checked out by the taxonomists Kirill Makarov, Andrey Matalin, Boris Kataev, Evgeniy Komarov, Dmitry Fedorenko and Igor Sokolov. After identification and counting, almost all specimens were disposed. Specimens of some species were dissected to determine the generative state. Some specimens of rare species were preserved and included in the private collection of Sergey Alexeev.

To describe and visualise carabid assemblages, we used non-metric multidimensional scaling based on Bray-Curtis Dissimilarity (qualitative), species number and Shannon Diversity Index. This data processing was performed in vegan R package (Oksanen et al. 2020).

### Step description

1. Sample plots were chosen in different kinds of urban habitats.
2. The beetles were sampled by pitfall traps during a whole season or, in some cases, a shorter period (1-3 months).
3. The beetles were identified and counted.
4. The dataset was compiled. This dataset includes raw data - the number of individuals sampled during the period between trap installation and the first sampling of two consecutive samplings. The relative abundance in units of ind./100 trap days were calculated as well.

Overall, investigations covered 13 seasons during a time span of 22 years. Unfortunately, we were not able to save all of the primary data. Therefore, we could not provide data on every sampling event for 17 surveys. For these, we have data summarised for the entire season. In such cases dwc: eventID and dwc: parentEventID are the same and sampling event means the whole season of sampling, which includes several actual events. In total, data on each sampling event are available for 41 surveys from 37 plots.

## Geographic coverage

### Description

The European part of Russia, Kaluga Oblast, Kaluga Urban Okrug, Kaluga City. The location of the sample plots was measured using Google Maps and Yandex Maps web services for plots established before 2003 and with satellite navigator (GPS) for ones studied later. Decimal degrees geographic coordinates are provided according to WGS 84 datum. Coordinates of sampling plots are available in Table 1.

### Coordinates

54.4808 and 54.5996 Latitude; 36.1965 and 36.3661 Longitude.

## Taxonomic coverage

### Description

Taxonomic coverage is given according to the GBIF Backbone Taxonomy (GBIF Secretariat 2011). This section of the Backbone derives from the Catalogue of Life (Anonymous 2011) and is curated by Wolfgang Lorenz (Lorenz 2021). TheCatalogue of Palearctic Coleoptera, compiled with the participation of several Russian carabidologists, was also used (Löbl and Löbl 2017).

In total, 189 species and 79091 specimens are included in this dataset. We identified one subspecies: *Harpalusxanthopuswinkleri* Schauberger, 1923, but since there are no other subspecies, we consider it as a species.

**Figure 10.**
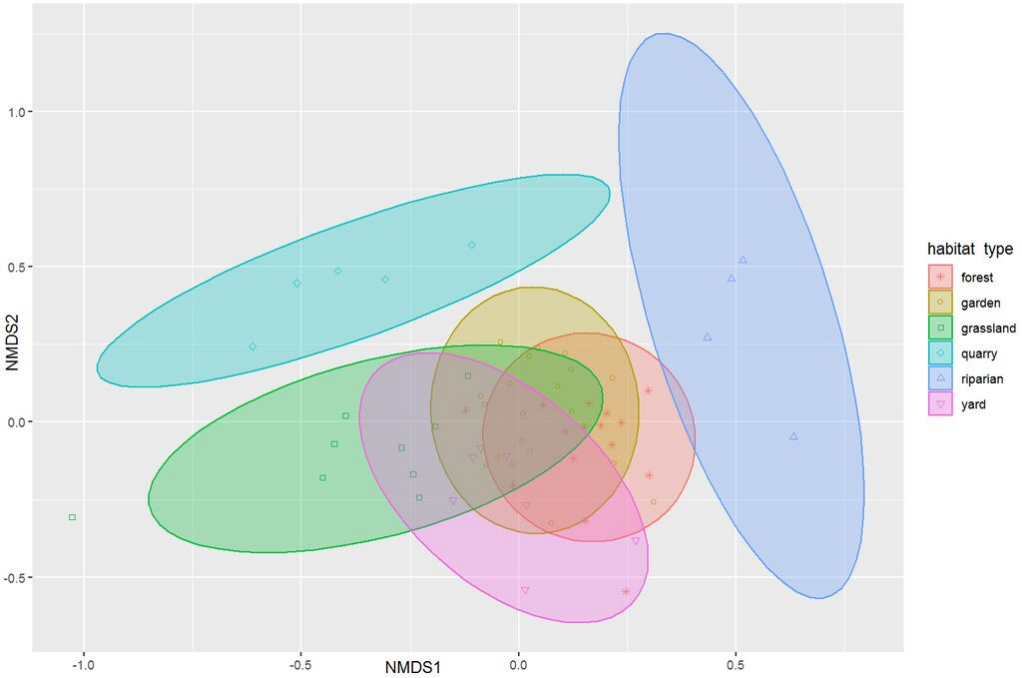
NMDS ordination graph (Bray-Curtis Dissimilarity)

In the NMDS ordination graph, two groups of samples are distinctly divided from samples of other types of habitats (Fig. 10). They are habitats of a former quarry and riparian habitats. Non-riparian forests, gardens and yards are not clearly distinguished from each other. Species richness (number of species) of surveys ranged between 24 and 84 species (Table 2). More species from diverse biotopes are riparian habitats and gardens.

This dataset contains most of the data on which the monograph "Inventory of the Ground Beetles (Coleoptera, Carabidae) of Kaluga Urban Okrug" (Aleksanov and Alexeev 2019) is based. In this book, we recorded 235 carabid species since 1994 to 2015. Four species were only caught in one suburban habitat and hence not included in this dataset because the list of species from this habitat is not completed yet. Other species were only collected by hand or window traps.

### Taxa included

**Table:** 

Rank	Scientific Name	Common Name
family	Carabidae	Ground beeles

## Usage licence

### Usage licence

Other

### IP rights notes

Attribution 4.0 International (CC BY 4.0)

## Data resources

### Data package title

Ground beetles (Carabidae) in urban habitats of Kaluga City (Russia)

### Resource link

https://www.gbif.org/dataset/5b4ba541-ad87-4d28-b8ca-a803335fd49d

### Alternative identifiers

http://gbif.ru:8080/ipt/resource?r=new_carabidae_kaluga_city1

### Number of data sets

1

### Data set 1.

#### Data set name

Ground beetles (Carabidae) in urban habitats of Kaluga City (Russia)

#### Data format

Darwin Core Archive format

#### Number of columns

25

#### Description

The dataset includes two related tables related by the eventID field – Events and Associated occurrences (Aleksanov and Alekseev 2021). The Event table consists of 13 fields, the Associated occurrences table - 12 fields. The occurrence table includes occurrence-present as well as occurrence-absent records.

**Data set 1.** 

Column label	Column description
eventID(Event Core, Occurrence Extension)	An identifier for the sample plot and the trapping period. https://dwc.tdwg.org/terms/#dwc:eventID A key field for relation between tables, categorical, 425 unique values,examples: "94-Park", "15-Bx-2015-09-12"
parentEventID (Event Core, Occurrence Extension)	An identifier for the sample plot. https://dwc.tdwg.org/terms/#dwc:parentEventID ID of season whole trapping period, in some cases eventID = parentEventID. Caregorical, 60 unique values, examples: "94-EBC", "97-Park", "08-Sev".
samplingProtocol (Event Core)	Sampling protocol. https://dwc.tdwg.org/terms/#dwc:samplingProtocol Textual description, constant: "soil pitfall traps"
samplingEffort (Event Core)	Amount of trap-days for each sampling term. https://dwc.tdwg.org/terms/#dwc:samplingEffort Textual description, example: "15 pitfall traps per 13 days"
habitat (Event Core)	Description of the habitat. https://dwc.tdwg.org/terms/#dwc:habitat Textual description, examples: "Garden including apple trees", "Grassy pine forest"
countryCode (Event Core)	The standard code for the Russian Federation according to ISO 3166-1-alpha-2. https://dwc.tdwg.org/terms/#dwc:countryCode Categorical, constant: "RU"
locality (Event Core)	The specific description of the place. https://dwc.tdwg.org/terms/#dwc:locality Brief textual description, 31 unique values, examples: "Kaluga city, Gagarina street", "Kaluga city, Berezujsky gully"
decimalLatitude (Event Core)	The geographic latitude in decimal degrees of the geographic centre of the data sampling place. https://dwc.tdwg.org/terms/#dwc:decimalLatitude Numerical variable of decimal type with a precision of 6 and scale of 4 ranged between 54.4808 and 54.5996
decimalLongitude (Event Core)	The geographic longitude in decimal degrees of the geographic centre of the data sampling place. https://dwc.tdwg.org/terms/#dwc:decimalLongitude Numerical variable of decimal type with a precision of 6 and scale of 4 ranged between 36.1965 and 36.3661
geodeticDatum (Event Core)	Spatial reference system (SRS) upon which the geographic coordinates are given in decimalLatitude and decimalLongitude are based. https://dwc.tdwg.org/terms/#dwc:geodeticDatum Categorical, constant: "WGS84"
coordinateUncertaintyInMetres (Event Core)	The maximum uncertainty distance in metres. https://dwc.tdwg.org/terms/#dwc:coordinateUncertaintyInMeters Numerical variable of integer type, constant: 50
eventDate(Event Core)	Trap period (YYYY-MM-DD/YYYY-MM-DD). https://dwc.tdwg.org/terms/#dwc:eventDate Date, 183 unique values, example: '2007-05-29/2007-06-05'
startDayOfYear (Event Core)	The earliest integer day of the year on which the Event occurred. http://rs.tdwg.org/dwc/terms/startDayOfYear Numerical, ranged between 97 and 282
endDayOfYear (Event Core)	The latest integer day of the year on which the Event occurred. http://rs.tdwg.org/dwc/terms/endDayOfYear Numerical, ranged between 118 and 315
occurrenceID (Occurrence Extension)	An identifier for the occurrence. https://dwc.tdwg.org/terms/#dwc:occurrenceID Numerical, integer counter with values between 1 and 84971
basisOfRecord (Occurrence Extension)	The specific nature of the record. https://dwc.tdwg.org/terms/#dwc:basisOfRecord Categorical according to vocabulary, constant: "HumanObservation"
scientificName (Occurrence Extension)	Scientific name according to GBIF Backbone. https://dwc.tdwg.org/terms/#dwc:scientificName Cathegorical based on checklist, example: "Amara spreta (Dejean, 1831)"
taxonRank (Occurrence Extension)	The taxonomic rank. https://dwc.tdwg.org/terms/#dwc:taxonRank Сategorical according to vocabulary, constant: "species"
occurrenceStatus (Occurrence Extension)	A statement about the presence or absence of this taxon in the trapping period. https://dwc.tdwg.org/terms/#dwc:occurrenceStatus Categorical according to vocabulary, "present" or "absent"
organismQuantity (Occurrence Extension)	The quantity of beetles. https://dwc.tdwg.org/terms/#dwc:organismQuantity Relative abundance expressed in values of decimal type. Numerical variable of decimal type, ranged between 0.02 and 242.222
organismQuantityType (Occurrence Extension)	The type of quantification system used for the quantity of beetles. https://dwc.tdwg.org/terms/#dwc:organismQuantityTypeTextual, constant: "individuals per 100 trap days"
kingdom (Occurrence Extension)	The full scientific name of the kingdom in which the taxon is classified. https://dwc.tdwg.org/terms/#dwc:kingdom Categorical according to GBIF Backbone checklist, constant: "Animalia"
individualCount (Occurrence Extension)	The number of individuals represented present at the time of the Occurrence. https://dwc.tdwg.org/terms/#dwc:individualCount Numerical of integer type, ranged between 0 and 1401
coordinatePrecision (Event core)	A decimal representation of the precision of the coordinates given in the decimalLatitude and decimalLongitude. http://rs.tdwg.org/dwc/terms/coordinatePrecision Numerical on decimal type, constant: 0.0001
georeferenceSources (Event core)	A list (concatenated and separated) of resources used to georeference the Location. http://rs.tdwg.org/dwc/terms/georeferenceSources Categorical, 2 unique values: "Google Maps" | "satellite navigation"

## Figures and Tables

**Table 1.** Brief description of the sites (sample plots) sampled during а period between 1994 and 2015. A brief explanation of the habitat types is given above. *Size of the open, unbuilt and undivided by roads, the area around sample plots facilitating dispersal of the ground beetles. This area can include various habitats, for example, woods, grasslands, gardens and others. Some plots are adjacent to each other so the open area around these has one and the same size. ** We define no areas which were larger than 1 km^2^ and belong to suburb landscapes or are aligned along rivers.

Type of habitat, land form	Coordinates (Latitude, Longitude)	Position in the city structure	Vegetation and land use (optional)	Size of unbuilt area around the plot, ha	Sampling period	Traps number	Plot ID (parent event)
Forest, watershed slope	54.5056 36.2469	city centre	Park. Old lime trees (*Tiliacordata*) with grassy lawns	15.5	20/04/1994 01/10/1994	30	94-Park
					20/04/1997 01/10/1997	30	97-Park
Forest, main river valley, S slope	54.5061 36.2436	city centre	Wood. Tree very dense layers dominated by box-elder (*Acernegundo*) and maple (*Acerplatanoides*) with ruderal weeds	15.5	18/05/2003 27/09/2003	15	03-Park
Forest, gully	54.5083 36.2447	city centre	Decidious wood dominated by box-elder (*Acernegundo*) and maple (*Acerplatanoides*) with ruderal weeds	14.97	20/04/1994 01/10/1994	30	94-Ber
					20/04/1997 01/10/1997	30	97-Ber
Forest, gully, bottom	54.5092 36.2458	city centre	Decidious wood dominated by box-elder (*Acernegundo*) and maple (*Acerplatanoides*) with ruderal weeds	14.97	03/05/2003- 27/09/2003	15	03-Ber
Forest, gully, E slope	54.5093 36.2455	city centre	Decidious wood dominated by lime (*Tiliacordata*), box-elder (*Acernegundo*), and maple (*Acerplatanoides*)	14.97	2004-05-02 2004-10-24	15	04-Ber
Forest, gully, E slope	54.5069 36.2678	city centre	Wood dominated by box-elder (*Acernegundo*) and maple (*Acerplatanoides*) with ruderal weeds	7.39	20/04/1994 01/10/1994	30	94-Zh
					20/04/1995 01/10/1995	30	95-Zh
Forest, gully, E slope	54.5081 36.2677	city centre	Wood dominated by box-elder (*Acernegundo*) and maple (*Acerplatanoides*) with ruderal weeds	7.39	03/05/2003 27/09/2003	15	03-Zh
Forest, gully, bottom	54.5064 36.2675	city centre	Wood dominated by the white willow (*Salixalba*) and poplars (*Populus* sp.) with the woolly burdock (*Arctiumtomentosum*), the Himalayan balsam (*Impatiensglandulifera*), and the stinging nettle (*Urticadioica*)	7.39	01/05/2011 22/10/2011	15	11-Zh
Forest, watershed slope	54.5069 36.3124	city periphery	Birch wood with tall mesophile herbs	18.35	23/04/2010 19/10/2010	10	10-Bb
Forest, watershed slope	54.5378 36.2542	city centre	Grassy pine forest	57.07	20/04/1994 01/10/1994	30	94-Kom
Forest, watershed slope	54.5381 36.2539	city centre	Pine forest with nemoral herbs and shrubs	57.07	20/04/1995 01/10/1995	30	95-Kom
Forest, gully, S slope	54.5382 36.2602	city centre	Broadleaved forest dominated by oak (*Quercusrobur*), maple (*Acerplatanoides*), and box-elder (*Acernegundo*)	57.07	18/05/2003- 27/09/2003	15	03-Kom
Riparian wooded habitat, main river valley, floodplain	54.4959 36.2641	city centre	Fringe of willow-woods (dominated by *Salixtriandra*) on the bank of Oka River, near the waters' edge, with grasses (*Bromusinermis* dominates), sedges and herbs (*Pentanemabritannicum*)	undefined	01/05/2015 15/10/2015	10	15-WW
Riparian wooded habitat, main river valley, floodplain	54.4965 36.2644	city centre	Box-elder (*Acernegundo*) spinney on the bank of Oka River, with sparse herb layer which consists of *Impatiensparviflora*, *Glechomahederacea*, in some sites *Urticadioica, Aegopodium podagraria*	undefined	01/05/2015 15/10/2015	10	15-Bx
Riparian wooded habitat, main river valley, floodplain	54.4985 36.3084	city centre	Willow-woods (dominated by *Salixtriandra*) on the bank of Oka River, in low site, with box-elder, *Rubus, Solanum, Urtica, Arctium*.	undefined	01/05/2015 15/10/2015	10	15-W1
Riparian wooded habitat, main river valley, floodplain	54.4985 36.3079	city centre	Willow-woods (dominated by *Salixtriandra*) on the bank of Oka River, in rather high site, withclosed crowns, with box-elder, *Urtica, Arctium*.	undefined	01/05/2015 15/10/2015	10	15-W2
Yard, watershed slope	54.5058 36.2853	city centre	Linear artificial wood dominated by maple (*Acerplatanoides*), box-elder (*Acernegundo*), ash (*Fraxinuspensylvanicus*), with tall ruderal herbs	1.72	05/06/2006 11/11/2006	12	06-Hosp
Yard, watershed slope	54.5082 36.2635	city centre	Yard consisting of flowerbeds and grass patches, surrounded by pavement and buildings	1.31	02/05/2003 27/09/2003	10	03-EBCp
					01/05/2004 20/10/2004	10	04-EBCp
					16/04/2007 26/10/2007	16	07-EBCp
Yard, watershed slope	54.5083 36.2742	city centre	Stands of trees between buildings in city centre. Dominated by the box-elder, Norway maple and green ash (*Fraxinuspennsylvanica*) with ruderal weeds	0.148	01/05/2011 22/10/2011	6	11-KSUt
Yard, watershed slope	54.5092 36.2733	city centre	Grassy yard between buildings in city centre. Dominated by the cat grass (*Dactylisglomerata*) and Kentucky bluegrass (*Poapratensis*), with sparse trees of birch and common pear (*Pyruscommunis*)	0.267	01/05/2011 22/10/2011	8	11-KSUh
Yard, watershed slope	54.5218 36.2612	city centre	Tree line dominated by lime (*Tiliacordata*) with lawns and buildings	0.44	25/05/2006 07/11/2006	15	06-TRC
Garden, watershed slope	54.4808 36.2554	suburb	Non-tilled garden including apple trees, currant shrubs, weeds.	undefined	27/05/2009 19/10/2009	15	09-N
Garden, tributary river valley	54.5081 36.3661	suburb	Garden consisting of apple trees, vegetable plot	undefined	28/04/1995 06/10/1995	12	95-Zhd
Garden, watershed slope	54.5087 36.2638	city centre	Garden plot with vegetables and decorative flowers and apple orchard in central part and with hedge from box-elder (*Acernegundo*) and common lilac (*Syringavulgaris*) and clump from the warty birch (*Betulapendula*), English oak (*Quercusrobur*), Norway maple (*Acerplatanoides*) and green ash (*Fraxinuspennsylvanica*) on the periphery	1.31	20/04/1995 11/10/1995	23	95-EBC
					02/05/2003 27/09/2003	15	03-EBCg
					01/05/2004 20/10/2004	15	04-EBCg
					2007-04-16 2007-10-26	26	07-EBCg
					01/05/2011 22/10/2011	15	11-EBCg
					18/04/2015 01/10/2015	15	15-EBCg
Garden, gully, NE slope	54.5158 36.2830	city centre	Moist garden consisting of apple trees, vegetable plot, grass patches	1.95	05/05/2006 11/11/2006	10	06-Pod
					20/04/2007 26/10/2007	12	07-Pod
					28/04/2009 20/10/2009	10	09-Pod
Garden, watershed slope	54.5250 36.2444	city periphery	Garden	3.58	07/04/1995 01/10/1995	20	95-Pz
Garden, watershed slope	54.5272 36.2867	city periphery	Poorly-drained garden. Most of its area is vegetable plot which is tilled many times a year. There are apple trees and currant shrubs.	30.02	02/05/2009 29/10/2009	15	09-Vet
Garden, watershed slope	54.5300 36.2550	city periphery	Garden	1.05	28/04/1995 04/07/1995	15	95-K
Garden, watershed slope	54.5708 36.3036	suburb	Garden	undefined	28/04/1995 04/07/1995	15	95-Olg
Garden, watershed slope	54.5724 36.2984	suburb	Garden including apple trees, currant shrubs, flowers, vegetables. Most of its area is tilled twice a year	undefined	01/05/2009 20/10/2009	13	09-Olg1
Garden, watershed slope	54.5753 36.2975	suburb	Non-tilled garden including apple trees, currant shrubs, weeds.	undefined	27/05/2009 19/10/2009	15	09-lg2
Garden, watershed slope	54.5968 36.2640	city periphery	School garden with sparse apple trees, flowers and mesotrophic and oligotrophic weeds	2.95	17/05/2008 01/11/2008	26	08-Sev
Garden, tributary river valley	54.5969 36.2348	city periphery	Garden including apple trees, currant shrubs, flowers, vegetables. Most of its area is tilled twice a year	undefined	29/04/1995 04/07/1995	15	95-Tim
					27/05/2009 19/10/2009	15	09-Tim
Grassland, watershed slope	54.5072 36.3141	city periphery	Grassland (fallow) with recent regeneration of birch (*Betulapendula*) on the site of abandoned field	18.35	23/04/2010 19/10/2010	10	10-BF
Grassland, watershed slope	54.5079 36.3147	city periphery	Meadow on a clay site disturbed by road construction activity dominated by wood small-reed (*Calamagrostisepigejos*) with tall herbs.	18.35	23/04/2010 19/10/2010	9	10-BC
Grassland, main river valley, S slope	54.5089 36.2333	city centre	Dry grassland on road embankment	0.44	01/06/2001 01/09/2001	4	01-Gag
Grassland, watershed slope	54.5290 36.2951	city periphery	Lawn between road and pavement dominated by cattail grass (*Phleumpratense*), cocksfoot (*Dactylus glomerata*) and legumes, with poplar trees	3.12	17/04/2007 25/10/2007	8	07-GrL
Grassland, watershed slope	54.5292 36.2972	city periphery	Railway bank of east-south-east exposition overgrown by *Bromusinermis, Vicia cracca* and other legumes and forbs	3.12	17/04/2007 25/10/2007	15	07-GrR
Grassland, watershed slope	54.5294 36.2967	city periphery	Linear site between railway and pavement, sometimes wet. Dominated by *Festucapratense* with significant contribution of *Centaureajacea*, *Medicagofalcata* and other Asteraceae and Fabaceae, locally dominated by *Calamagrostisepigejos*, locally with sparse poplar trees	3.12	17/04/2007 25/10/2007	12	07-Gr
Grassland, watershed slope	54.5473 36.3147	city periphery	Mesophile meadow dominated by randall (*Festucapratense*), cocksfoot (*Dactylus glomerata*) and lady's-mantle (*Alchemilla*), with hygrophilic herbs	6.48	08/06/2006 07/11/2006	13	06-O
Grassland, tributary river valley	54.5918 36.2267	city periphery	Tall-grass meadow on railway embankment near river and forest	undefined	05/06/2006 11/08/2006	15	06-Sil2
Grassland, tributary river valley	54.5929 36.2266	city periphery	Tall-grass meadow on railway embankment near river and forest	undefined	05/06/2006 11/08/2006	15	06-Sil1
Former quarry, watershed slope	54.5966 36.1992	suburb	Calcareous rocky outcrops with single willows and sea-buckthorns	undefined	19/04/2010 23/10/2010	10	10-MR
Former quarry, watershed slope	54.5988 36.2036	suburb	Grassland dominated by *Calamagrostisepigeios* in open-pit bottom	undefined	19/04/2010 23/10/2010	10	10-MG
Former quarry, watershed slope	54.5992 36.1981	suburb	Young site of open-pit bottom with pond and willow-shrub.	undefined	19/04/2010 23/10/2010	10	10-MP
Former quarry, watershed slope	54.5995 36.1993	suburb	Grove dominated by pine, birch, willows and sea-buckthorn.	undefined	19/04/2010 23/10/2010	10	10-MW
Former quarry, watershed slope	54.5996 36.1965	suburb	Open-pit side with clay soils overgrown with legumes and forbs herb layer	undefined	19/04/2010 23/10/2010	10	10-MC

**Table 2.** Descriptive characteristics of carabid samples for different types of habitats in the City of Kaluga.

Group	Sample (plotby year) number	Total species	Species number			Shannon Index		
			median	min	max	median	min	max
Forest	15	130	51.0	28	84	2.58	1.73	2.81
Riparian wooded habitat	4	99	51.5	33	83	2.45	1.90	3.18
Yard	7	88	38.0	24	54	2.48	2.20	3.11
Garden	20	149	53.5	34	65	2.82	2.08	3.19
Grassland	9	115	46.0	27	56	2.58	2.04	2.94
Quarry	5	191	42.0	33	56	2.43	2.33	2.98

**Table 3.** Summary of sampling seasons

ID (parent event)	individuals count	number of species	duration, days	sampling efforts, 100 trap days	relative abundance, ex/100 trap days
94-Park	2234	54	164	49.2	45.4
97-Park	2266	60	164	49.2	46.1
03-Park	1148	29	124	18.6	61.7
94-Ber	1513	44	164	49.2	30.8
97-Ber	1774	51	164	49.2	36.1
03-Ber	1009	52	138	20.7	48.7
04-Ber	579	28	169	25.35	22.8
94-Zh	1099	63	164	49.2	22.3
95-Zh	2164	65	164	49.2	44
03-Zh	1103	46	139	20.85	52.9
11-Zh	918	36	160	24.15	38
10-Bb	1788	46	171	16.3	109.7
94-Kom	1891	63	164	49.2	38.4
95-Kom	1599	65	164	49.2	32.5
03-Kom	976	39	125	18.75	52.1
15-WW	1557	83	156	14.49	107.5
15-Bx	1100	33	156	15.6	70.5
15-W1	2731	49	143	13.19	207.1
15-W2	2269	54	156	15.04	150.9
06-Hosp	647	34	154	18.48	35
03-EBCp	999	49	148	14.8	67.5
04-EBCp	841	51	165	16.5	51
07-EBCp	999	54	182	28.41	35.2
11-KSUt	471	24	160	9.6	49.1
11-KSUh	1031	34	160	12.8	80.5
06-TRC	1115	38	160	24	46.5
09-N	917	34	114	17.1	53.6
95-Zhd	1662	42	43	5.16	322.1
95-EBC	6729	84	174	40.02	168.1
03-EBCg	2147	57	148	22.2	96.7
04-EBCg	885	51	142	21.3	41.5
07-EBCg	2120	60	182	47.93	44.2
11-EBCg	864	49	160	24	36
15-EBCg	246	39	155	23.25	10.6
06-Pod	1377	56	183	18.3	75.2
07-Pod	2510	63	180	21.94	114.4
09-Pod	1251	61	167	16.7	74.9
95-Pz	1343	44	177	35.4	37.9
09-Vet	2202	54	171	25.26	87.2
95-K	842	39	67	10.05	83.8
95-Olg	2008	45	67	10.05	199.8
09-Olg1	1287	55	163	20.32	63.3
09-lg2	1114	43	123	18.45	60.4
08-Sev	2037	64	163	41.36	49.3
95-Tim	1984	64	66	9.9	200.4
09-Tim	999	53	138	20.7	48.3
10-BF	958	51	171	16.88	56.8
10-BC	583	56	171	15.03	38.8
01-Gag	627	27	92	3.68	170.4
07-GrL	267	38	166	13.39	19.9
07-GrR	338	47	181	26.55	12.7
07-Gr	693	45	181	22.65	30.6
06-O	830	46	147	19.11	43.4
06-Sil2	543	46	67	10.05	54
06-Sil1	1377	51	67	10.05	137
10-MR	223	38	178	17.8	12.5
10-MG	697	42	162	16.2	43
10-MP	863	56	178	17.8	48.5
10-MW	276	33	178	17.8	15.5
10-MC	471	43	178	17.8	26.5
